# The Content of Phenolic Compounds and Radical Scavenging Activity Varies with Carrot Origin and Root Color

**DOI:** 10.1007/s11130-013-0351-3

**Published:** 2013-04-24

**Authors:** Maria Leja, Iwona Kamińska, Maike Kramer, Anna Maksylewicz-Kaul, Dietmar Kammerer, Reinhold Carle, Rafal Baranski

**Affiliations:** 1 Department of Botany and Plant Physiology, Faculty of Horticulture, University of Agriculture in Krakow, 29 Listopada 54, 31-425 Kraków, Poland; 2 Department of Plant Cytology and Embryology, Institute of Botany, Jagiellonian University, Grodzka 52, 31-044 Kraków, Poland; 3 Plant Foodstuff Technology, Institute of Food Science and Biotechnology, Hohenheim University, Garbenstraße 25, 70599 Stuttgart, Germany; 4 Department of Genetics, Plant Breeding and Seed Science, Faculty of Horticulture, University of Agriculture in Krakow, 29 Listopada 54, 31-425 Kraków, Poland

**Keywords:** Antioxidant activity, DPPH, Folin-Ciocalteu assay, Genetic diversity, Phenolics, RSA

## Abstract

The contents of phenolic compounds and radical scavenging activities were assessed in a carrot collection comprising 35 cultivars, landraces and breeding populations. The accessions originated from various world regions and they represented Eastern and Western carrot gene pools. In two-year field trial carrot roots of orange, red, yellow, white and purple color were cultivated, freeze-dried and analyzed for phenolic content by Folin-Ciocalteu assay and UV/Vis assay. Radical scavenging activity in the extracts was determined with a stable DPPH radical. Carrots developing purple roots possessed on average 9 times more phenolics than roots of other colors. Furthermore, they were rich in anthocyanins that caused very high antiradical activity. Red carrots showed higher antioxidant activity than orange, yellow and white carrots and in the season of lower rainfall they accumulated higher amounts of phenolic compounds. Carrots of Asian origin belonging to Eastern gene pool were more often purple or red and richer in phenolics and had higher antiradical activity than those from the Western gene pool with mainly orange roots.

**Electronic supplementary material:**

The online version of this article (doi:10.1007/s11130-013-0351-3) contains supplementary material, which is available to authorized users.

## Introduction

Fruits and vegetables are valuable sources of health-promoting substances active in neutralization of reactive oxygen species. Among them carrot (*Daucus carota* L.) belongs to horticultural crops of high recognition and economic importance due to its nutritional value and high concentration of bioactive constituents.

Cultivated carrots originated in the Afghanistan region and were yellow and purple. From this center of domestication carrots were grown as a root crop to the East and West with the incorporation of several characteristics contrasting those two geographic regions. The Eastern carrot spread to central and north Asia and then to Japan. Red colored carrot is typical for India and also was introduced to Japan. In contrast, Western carrot type is characterized initially by yellow and later by orange root color. This carrot type spread to West and now dominates in Europe and America [1]. Carrot is rich in pro-healthy antioxidants both of lipophylic (carotenoids) and hydrophilic (phenolic compounds) characters. Although carotenoid content varies considerably among carrot genotypes [2], usually orange carrots contain high amounts of α- and β-carotene; yellow carrots contain lutein, the red color of carrots is due to lycopene, while polyphenol substances, mostly anthocyanins are typical for purple roots [3, 4].

The role of carrot carotenoids as the precursors of vitamin A as well as excellent antioxidants has been commonly known for many years [5–7]; however, phenolic compounds and their antioxidant capacity in *Daucus* cultivars have been also investigated recently. According to Arscott and Tanumihardjo [4] carrot contains phenolic constituents with a single aromatic ring (phenolic acids), mainly chlorogenic acid. Zhang and Hamauzu [8] found that carrots contained mainly hydroxycinnamic acid derivatives, among them chlorogenic acid represented 42.2–61.8 % of total phenolics. Chlorogenic acid is also accumulated in baby carrot roots [9], this compound accompanied by ferulic and dicaffeoylquinic acid reached 82 % of total phenolics in wounded root tissue [10]. Chlorogenic acid, caffeic acid, *p*-hydroxybenzoic acid, ferulic acid and other cinnamic acid isomers predominated in carrots of different colors [3]. Alasalvar et al. [11] identified 11 different phenolic acids in orange, purple, yellow and white carrots, and the total concentration of all phenolic acids was the highest in purple carrots.

High nutritional value of food, *inter alia*, is due to the presence of compounds exhibiting antioxidant activity. *D*. *carota* tissue possesses such compounds active in both lipophilic (carotenes, xanthophylls) and hydrophilic (polyphenols) cell fractions. Phenolic compounds have reducing properties as they are hydrogen- or electron-donating agents [12]. High ability of phenolics to neutralize radicals results from their chemical structure; the higher the number of hydroxyl groups [13] (especially those bonded to the B-ring) and double bonds in the molecule [14], the greater the ability to scavenge radicals. Phenolic compounds contribute significantly to antioxidant properties of plant extracts, which is often demonstrated by high correlation between the level of phenolics in the tissue and antiradical activity of the extract [15–17].

Up until now the assessments of carrot cultivars with regard to the presence of phenolic compounds have been performed using low number of cultivars (sometimes of unknown origin [18]) and often restricted to only individual accession of a given root color or location [19]. However, the genetic background is a critical factor determining qualitative and quantitative chemical composition of plant tissue, thus the results obtained in different research projects are often not congruent to each other. These discrepancies are frequently exaggerated by the use of different extraction and analytical methods. For example, the content of anthocyanins in various reports ranged from 100 to 243 mg 100 g^−1^ FW in purple carrots and from 16 to 71 mg 100 g^−1^ FW in orange carrot [3, 11, 20, 21]. Even more profound differences concern carotenoids, which particularly in purple carrots ranged from 0.5 mg to 17.4 mg 100 g^−1^ FW [21, 22]. Additionally, the compound concentration may considerably change across root section depending on tissue type and cell age as it was demonstrated by mapping carotenoid distribution in carrot roots using Raman spectroscopy [23]. Thus, the aim of the present research was to evaluate a broad spectrum of carrot commercial cultivars, breeding populations and landraces as the source of phenolic compounds and to assess differences between the accessions of various root color and origin.

## Materials and Methods

### Plant Material and Field Trial Conditions

Thirty five carrot cultivars, landraces and breeding lines of various origins were grown in 2009 and 2010 in field trials near Krakow in Poland (Online Resource 1). The trials comprised 15 accessions developing orange roots, eight yellow, five white, five red and two purple roots. The accessions were classified either as Western or Eastern carrot type based on the previous results of molecular assessment using simple sequence repeat loci [24]. In case of the accessions, which were not included in the molecular assessment, the classification was performed based on the root color and information on the accession origin.

Plants were cultivated on loess soil with humus. Air and soil temperature during the growing period were similar in both years of cultivation (mean values of 2 years: 17.5 and 18.3 °C for air and soil, respectively). Total rainfalls in 2009 were lower than in 2010 (178.3 and 451.2 mm, respectively). The routine techniques of cultivation and plant protection, recommended for carrot crop production were applied. Plots were arranged in a randomized block design with four replications.

### Chemical Analyses

Analyses of phenolic compounds and antioxidant activity were performed using 1 kg of healthy, intact roots from each field replication. Approx. 10–15 roots (per field replication) were cut, freeze-dried, milled and stored until analyzed.

#### Phenolic Compounds

The contents of total phenolic compounds as well as groups of phenolic constituents such as phenylpropanoids (derivatives of cinnamic acid), flavonols and anthocyanins were determined by measurement of UV/Vis absorbance according to Fukumoto and Mazza [25]. Reaction mixture consisted of 0.25 ml of 80 % methanol extract of freeze-dried tissue with 0.25 ml of 0.1 % HCl in 96 % ethanol and 4.5 ml of 2 % HCl. The absorbance was measured at 280 nm using chlorogenic acid as a standard, 320 nm (caffeic acid as standard) and 360 nm (quercetin as standard) for total phenols, phenylpropanoids and flavonols, respectively. Absorbance of anthocyanins content was read at 520 nm and expressed as cyanidin, according to its molar extinction.

Additionally, the content of total phenols was determined colorimetrically with Folin’s reagent according to Singleton et al. [26]. For this purpose, 0.25 g of freeze-dried tissue was extracted with 10 ml of acetone/water (7:3, *v*/*v*) under stirring (ambient temperature, 320 rpm) for 30 min. After filtration, the material was re-extracted in the same manner using 10 ml of ethanol/water (4:1, *v*/*v*). The combined supernatants were evaporated to dryness *in vacuo* at 30 °C, and the residue was dissolved in 15 ml of deionised water. Aliquots of 50 μl of the diluted extracts or water as sample blank were mixed with 60 μl of the Folin–Ciocalteu reagent (diluted with deionised water, 1:6, *v*/*v*) in microplates for spectrophotometric determinations. After 3 min, 80 μl of a 7.5 % Na_2_CO_3_ solution (*w*/*v*) was added and incubated after thorough stirring at 20 °C in the dark. After 60 min, absorbance was read using a Power Wave microplate spectrophotometer at a detection wavelength of 760 nm. Gallic acid was used as the reference compound for calibration.

#### Radical Scavenging Activity

Radical scavenging activity (RSA) was determined using a stable DPPH radical and expressed as the percentage of the radical neutralization after 30 min incubation [27]. The ethanolic solution of DPPH^•^ (0.1 mM) was used as a point of reference for monitoring the decrease of its absorbance at 516 nm after addition of tissue extract. The control was prepared with 80 % methanol instead of carrot root extract. The concentration of freeze-dried tissue in the reaction mixture was 0.04 %. Only in case of purple carrots the reaction mixture was diluted twice to get 0.02 % final concentration of plant tissue.

### Statistics

The measurements for Folin–Ciocalteu assay were performed in two replications; all other measurements were done in four replications. The results were statistically verified using analysis of variance and the significance of differences between the means was tested using Newman–Keuls test or *t*-test at the significance level *p* = 0.05. Correlations between compound content and RSA were assessed using Pearson linear correlation coefficient.

## Results and Discussion

The assessment of phenolic compounds content and antiradical activity revealed, in general, no significant (*p* > 0.05) differences between the two experimental years except of the total phenolics content determined in Folin–Ciocalteu assay, which was 1.5 times higher in 2009 than in 2010 (*p* = 0.016). This difference was mainly caused by higher content of phenolics in 2009 found in accessions developing red roots while the remaining accessions of white, yellow, orange and purple roots had similar level of phenolics and antiradical activity in both years.

### Effect of Carrot Root Color

#### Phenolic Compounds

The content of phenolic compounds in the accessions evaluated varied considerably from 19.8 to 342.2 mg 100 g^−1^ FW as determined spectrophotometrically in UV/Vis range (Table 1). Purple roots possessed, on average, almost nine times more phenolics than roots of other colors, 311.5 and 34.9 mg 100 g^−1^ FW, respectively. Among accessions developing roots other than purple, variation of phenolic content was much lower and ranged from 19.8 to 61.9 mg 100 g^−1^ FW. White roots usually had the least phenolics; almost two-fold less than orange or red roots (excepting ‘Mestnaya’ cultivar, with white flesh and purple skin) (Table 2).

**Table 1:** The content of phenolic compounds [mg 100 g^−1^ FW] and RSA [%] in carrot extracts; means ± standard error

Root color	Accession name	Type^a^	Total phenols (Folin–Ciocalteu)^b^	Total phenols (UV/Vis)	Phenyl-propanoids	Flavonols	Anthocyanins	RSA
White	Blanche 1/2 longue des vosges	W	16.8 ± 0.7	23.4 ± 2.1	5.3 ± 0.5	3.4 ± 0.4	0.2 ± 0.03	5.4 ± 0.6
	Kuettiger	W	14.0 ± 0.7	22.8 ± 0.7	4.5 ± 0.1	3.4 ± 0.1	0.2 ± 0.00	3.6 ± 0.4
	Mestnaya	E	29.4 ± 2.5	33.5 ± 3.4	7.6 ± 0.8	4.5 ± 0.5	0.3 ± 0.03	10.6 ± 0.4
	White Belgian	W	19.0 ± 1.1	21.4 ± 1.4	5.2 ± 0.3	3.3 ± 0.3	0.2 ± 0.04	6.8 ± 1.3
	White Satin	W	10.8 ± 1.3	19.8 ± 1.2	4.3 ± 0.3	2.8 ± 0.2	0.1 ± 0.02	3.7 ± 0.4
Yellow	BL-710015	W	10.5 ± 0.8	29.0 ± 1.0	6.0 ± 0.3	4.7 ± 0.2	0.4 ± 0.04	3.6 ± 0.8
	China Yellow	E	14.3 ± 0.9	30.6 ± 2.9	6.0 ± 0.7	5.2 ± 0.5	0.4 ± 0.05	3.8 ± 1.5
	Lobbericher	W	21.1 ± 0.5	35.1 ± 1.7	7.6 ± 0.4	5.5 ± 0.3	0.4 ± 0.04	5.7 ± 0.6
	Mestnaya-S1-p	E	43.6 ± 1.4	42.2 ± 2.0	9.5 ± 0.6	6.7 ± 0.3	0.5 ± 0.02	8.6 ± 0.8
	Mestnaya-S1-y	E	24.6 ± 1.6	36.1 ± 3.4	7.3 ± 0.8	5.3 ± 0.6	0.4 ± 0.05	6.3 ± 0.9
	Shima Ninjin	E	22.4 ± 0.7	38.9 ± 3.2	9.3 ± 0.8	6.7 ± 0.2	0.5 ± 0.06	8.7 ± 3.0
	Yellow Belgian	W	19.9 ± 0.4	38.0 ± 2.6	8.1 ± 0.4	5.9 ± 0.5	0.5 ± 0.05	5.6 ± 0.6
	Yellowstone	E	15.5 ± 1.5	34.4 ± 1.6	7.2 ± 0.3	5.5 ± 0.3	0.5 ± 0.03	5.4 ± 0.7
Orange	Amsterdam 3	W	23.6 ± 1.4	37.6 ± 2.4	8.5 ± 0.8	6.5 ± 0.6	0.5 ± 0.07	5.1 ± 0.5
	BL-CR2-6032	W	28.5 ± 1.1	34.7 ± 1.9	8.5 ± 0.5	5.9 ± 0.6	0.4 ± 0.09	5.8 ± 1.3
	BL-JKI-4	E	53.8 ± 4.4	53.3 ± 2.9	13.6 ± 1.1	8.5 ± 0.5	0.7 ± 0.04	9.3 ± 0.7
	BL-JKI-5	E	58.6 ± 2.6	61.9 ± 3.4	16.6 ± 1.0	9.9 ± 0.6	0.7 ± 0.04	13.7 ± 0.6
	Finezja	W	20.9 ± 1.2	39.2 ± 2.3	8.6 ± 0.6	7.0 ± 0.6	0.6 ± 0.07	3.5 ± 0.8
	Garga Serk	E	26.4 ± 0.9	33.5 ± 1.3	6.9 ± 0.4	4.9 ± 0.3	0.4 ± 0.03	5.5 ± 0.8
	Hekinan Senko 5sun	W	18.7 ± 1.2	32.6 ± 2.1	7.5 ± 0.6	5.7 ± 0.4	0.4 ± 0.04	3.9 ± 0.6
	Kokubu Senko Oonaga	E	33.8 ± 2.2	51.5 ± 5.1	11.9 ± 1.0	8.6 ± 1.0	0.7 ± 0.13	8.1 ± 1.2
	Long Red	E	25.8 ± 0.2	35.4 ± 0.8	8.9 ± 0.2	5.6 ± 0.2	0.4 ± 0.01	6.6 ± 0.6
	Nantes Apollo	W	21.2 ± 0.4	30.7 ± 1.0	7.2 ± 0.3	5.0 ± 0.3	0.4 ± 0.03	4.0 ± 0.8
	Nerac	W	23.2 ± 0.7	36.4 ± 1.9	8.8 ± 0.4	6.1 ± 0.4	0.4 ± 0.05	6.2 ± 0.6
	Red Carrot	E	22.8 ± 0.3	29.9 ± 1.6	6.8 ± 0.4	4.7 ± 0.3	0.4 ± 0.02	6.8 ± 0.5
	Santa Cruz	W	26.1 ± 1.4	41.2 ± 1.7	10.4 ± 0.5	6.7 ± 0.3	0.5 ± 0.04	6.8 ± 0.7
	Sapporo Futo	W	26.5 ± 1.3	43.8 ± 2.9	10.0 ± 0.6	7.5 ± 0.6	0.6 ± 0.05	6.2 ± 0.8
	Stratova	W	29.7 ± 2.2	32.3 ± 1.8	8.0 ± 0.8	5.3 ± 0.4	0.4 ± 0.01	7.8 ± 1.0
Red	Gajar	E	38.4 ± 5.1	43.1 ± 3.6	11.4 ± 1.6	6.9 ± 0.8	0.4 ± 0.05	12.8 ± 2.2
	Kintoki	E	22.1 ± 1.9	40.0 ± 3.1	8.5 ± 0.6	6.7 ± 0.4	0.6 ± 0.04	5.1 ± 0.5
	Nutrired	E	24.5 ± 1.7	35.9 ± 1.5	7.6 ± 0.4	5.9 ± 0.3	0.5 ± 0.03	5.8 ± 0.7
	Pusa Kesar	E	32.3 ± 4.6	37.8 ± 3.0	9.1 ± 1.2	5.9 ± 0.5	0.4 ± 0.03	11.0 ± 2.4
	Shahpur Special	E	37.7 ± 5.2	45.2 ± 4.6	11.6 ± 2.0	7.3 ± 1.0	0.5 ± 0.03	12.0 ± 2.7
Mean values of non-purple accessions			25.0	34.9	8.0	5.6	0.4	5.5
Purple	Anthonina	W	267.1 ± 19.2	342.2 ± 25.6	102.6 ± 8.5	56.4 ± 4.6	74.0 ± 6.58	53.1 ± 5.9
	Deep purple	E	224.3 ± 21.6	280.8 ± 23.1	87.4 ± 8.5	46.7 ± 3.8	55.8 ± 5.29	49.5 ± 6.5
Mean values for purple accessions			245.7	311.5	95.0	51.6	64.9	51.3

^a^Type: Accessions were classified to either the Eastern (E) or Western (W) type according to DNA analysis of simple sequence repeats (Baranski, Maksylewicz-Kaul, Nothnagel, Cavagnaro, Simon & Grzebelus, 2012) or the accession origin

^b^For Folin–Ciocalteu assay the content of total phenols is expressed in mg gallic acid per 100 g FW

**Table 2:** The content of phenolic compounds [mg 100 g^−1^ FW] and RSA [%] depending on carrot root color

Root color	Total phenols (Folin–Ciocalteu)	Total phenols (UV/Vis)	Phenylpropanoids	Flavonols	Anthocyanins	RSA
White	18.0 a^a^	24.2 a	5.4 a	3.5 a	0.2 a	6.0 a
Yellow	21.5 a	35.5 ab	7.6 a	5.7 b	0.5 a	6.0 a
Orange	29.3 ab	39.6 b	9.5 a	6.5 b	0.5 a	6.6 a
Red	31.0 b	40.4 b	9.6 a	6.5 b	0.5 a	9.3 b
Purple	245.7 c	311.5 c	95.0 b	51.6 c	64.9 b	51.3 c

^a^Means followed by the same letter in each column do not differ significantly according to Newman–Keuls test at *p* = 0.05

The contents of total phenols determined in Folin–Ciocalteu assay were, on average, lower by 21–41 % depending on root color, but the results of both assays were highly congruent as the correlation coefficient was *r* = 0.99. Lower values obtained in Folin–Ciocalteu assay can result from the fact that they were expressed in units of gallic acid used as the standard while in direct measurements of phenol absorbance values were expressed in units of chlorogenic acid standard. Measurements of phenol absorption in the UV/Vis assay can be biased by the presence of compounds other than phenols that also absorb at 280 nm wavelength. Additionally, both assays required different extraction procedures.

The significant differences between accessions were also observed in the contents of individual groups of phenolic compounds. Similarly, as in the case of total phenols, the highest levels of cinnamic acid derivatives (phenylpropanoids), flavonols and anthocyanins were found in purple roots while white carrots contained on average the lowest amounts of these compounds (Table 3). Among non-purple accessions, those developing orange or red roots had similar content of the listed compounds (Table 2) and were richer in phenylpropanoids than accessions with yellow and white roots by 26 and 77 %, respectively, and were also richer in flavonols by 14 and 86 %, respectively. Although the observed differences were large, they seem to be of low importance due to the fact that the contents of phenylpropanoids and flavonols were low (8.1 and 5.6 mg 100 g^−1^ FW, respectively). Non-purple roots were also almost free of anthocyanins (0.1–0.7 mg 100 g^−1^ FW). In contrast, purple roots were very rich in anthocyanins and possessed on average 64.9 mg 100 g^−1^ FW and additionally they had 14 and 8 times more phenylpropanoids and flavonols, respectively, than roots of other colors. ‘Deep Purple’, a modern F1 hybrid cultivar, had 55.8 mg anthocyanins in 100 g^−1^ FW that placed it in a group of the most anthocyanin rich vegetables and fruits [28]. Another cultivar evaluated, ‘Anthonina’, was even richer than ‘Deep Purple’ by over 30 %.

**Table 3:** Contribution of phenolic compounds [%] to total phenols depending on root color

Root color	Phenylpropanoids	Flavonols	Anthocyanins
White	22.3 a^a^	14.5 a	0.8 a
Yellow	21.4 a	16.0 b	1.4 b
Orange	24.0 a	16.4 b	1.3 b
Red	23.8 a	16.1 b	1.2 b
Purple	30.5 b	16.6 b	20.8 c

^a^Means followed by the same letter in each column do not differ significantly according to Newman–Keuls test at *p* = 0.05

In the literature, the content of anthocyanin pigments ranged from 0 to 350 mg 100 g^−1^ FW in orange and dark purple carrots [4]. According to a report from Lazcano et al. [29] purple carrots contained 38–98 mg 100 g^−1^ FW of total anthocyanins, which is similar to data presented in this paper. Even a higher level (208–243 mg 100 g^−1^ FW) was reported by Kammerer et al. [20].

The main pigment identified in purple carrots was cyanidin-3-sinapoylxylosyl-glucose [29]. Kammerer et al. [30] detected in purple carrots pelargonidin and peonidin glycosides. It should be emphasized that carrot roots exhibit often different colors depending on the tissue; thus, color seen in a root cross section is heterogeneous. Particularly the phloem (root flesh) can differ from the xylem (core). Cyanidin-3,2-xylose galactoside dominated in purple-yellow carrot and cyanidin-3,2-xylose galactoside-glucose dominated in purple-orange. However, it is not clear if different composition is related to tissue color or to the fact that the authors used different carrot cultivars. Total anthocyanin level measured in purple-yellow and purple-orange roots was about 100 mg 100 g^−1^ FW [3].

The contribution of groups of phenolic compounds to total phenols was hardly dependent on root color (Table 3). Phenylpropanoids accounted for 21.4–24 % of total phenols and only in purple roots they were more abundant (30.5 %). The share of flavonols was about 16 % independently on root color except white roots that had slightly less flavonols (14.5 %). Low amounts of flavonoids contradict findings of Yen et al. [31] who determined the concentration of total flavonoids (including probably anthocyanins) in red carrot cubes at the similar level to that of total phenols. Anthocyanins occurred in considerable amounts in purple roots only (20.8 %) while in other roots their presence was below 2 %. Thus, on average, the ratio of phenylpropanoids to flavonols to anthocyanins was 30:16:23 and 22:16:1 in purple and non-purple roots, respectively.

#### Antiradical Activity

Radical scavenging activity (RSA) varied considerably among accessions. In general, RSA was relatively low and only in seven accessions exceeded 10 % of free radical neutralization (Table 1). High antiradical activity (about 50 % of DPPH^•^ scavenging) was observed for purple root extracts. The RSA values for purple roots remained underestimated as tissue concentration in the extract was two times lower than in extracts from roots of other colors. High antiradical activity of purple-yellow and purple-orange carrots was previously reported by Sun et al. [3], who measured such activity by DPPH and ABTS methods. Also Gajewski et al. [32] found higher antioxidant capacity in methanolic extracts from purple carrots than in extracts from orange and yellow carrots. In the present work, orange, white and yellow roots exhibited RSA, on average, at 6 % level and only red roots had higher activity (9.3 %) (Table 2). Only Yen et al. [31] observed very high, reaching even 80–98 % of DPPH^•^ neutralization activity in red carrot roots; however, those authors used very high (2–20 mg DM cm^−3^ of extract) tissue concentration.

Antiradical activity depended on the content of phenolic compounds. Very high correlation (*r* > 0.95) was found between RSA and total phenols, as well as phenylpropanoids, flavonols and anthocyanins (Table 4). Such high correlations were mainly due to high concentration of anthocyanins present in purple roots. This finding was supported by correlation coefficients when purple roots were excluded from calculations. In such case, the correlation between RSA and anthocyanins decreased to a low value (0.32) as there were almost no anthocyanins in orange, red, white and yellow roots. Phenylpropanoids seemed to be the most responsible for antiradical activity (*r* = 0.74) while flavonols showed intermediate correlation.

**Table 4:** Correlation coefficients between RSA and the content of phenolic compounds. Correlation coefficients calculated for data with purple carrots excluded are given in brackets

	RSA	Total phenols (Folin–Ciocalteu)	Total phenols (UV/Vis)	Phenylpropanoids	Flavonols
Total phenols (Folin–Ciocalteu)	0.99 (0.82)				
Total phenols (UV/Vis)	0.98 (0.64)	0.99 (0.83)			
Phenylpropanoids	0.98 (0.74)	0.99 (0.89)	1.00 (0.97)		
Flavonols	0.98 (0.54)	0.99 (0.76)	1.00 (0.98)	1.00 (0.94)	
Anthocyanins	0.96 (0.32)	0.98 (0.62)	0.99 (0.90)	0.99 (0.81)	0.99 (0.95)

The results obtained are congruent with the report that antioxidant capacity of hydrophilic extracts of carrot roots correlated with the total phenol content [33]. Chlorogenic acid, total phenols and total anthocyanins significantly correlated with total antioxidant capacity measured using DPPH and ABTS methods in hydrophilic phase extracts from root of different colors [3]. According to Zhang and Hamauzu [8] all phenolic extracts had higher RSA than pure chlorogenic acid, ascorbic acid and β-carotene. It was also reported that hydrophilic extracts had much higher antioxidant capacity than hydrophobic ones; in the case of purple-yellow carrots the difference was even 90 times [3]. According to those data, mainly phenolic substances seem to be responsible for antiradical system in carrot roots.

### Effect of Carrot Gene Pool

In the present work, carrot accessions originating from various world regions were compared. In particular, the experiment comprised 14 accessions from Europe, 16 from Asia (including five from Japan), four from USA and one from Ethiopia. The classification of carrot accession to either Western or Eastern gene pool, done previously by Baranski et al. [24], was based on the polymorphism of DNA simple sequence repeat loci. For accessions with no molecular assessment available, additional criteria were set to classify them to one of these two types. As orange and white root color are characteristic for Western carrot type, the presence of other color and Asian origin was chosen as criteria to include an accession to Eastern type. Thus Western and Eastern carrot types were finally represented by 17 and 18 accessions, respectively (Online Resource 1). It should be stressed that the lack of clear delimitation between Eastern and Western types depending on the country of carrot origin or root color is the result of an intensive and long lasting breeding in which materials of various origins were crossed. For example, European materials were used to obtain cultivars in Japan while Japanese cultivars were used as parental populations in American breeding programs.

The comparison of Western and Eastern carrot types with regard to the content of phenolic compounds and RSA could be biased by the presence of only two purple accessions, which as shown above, possessed a considerably higher level of phenolics and higher antiradical capacity. Therefore, purple accessions were excluded from further comparisons of both carrot types.

Eastern carrots had higher content of phenolic compounds than Western carrots by 53.2 and 24.1 % measured in Folin–Ciocalteu and UV/Vis assays, respectively (Table 5). The amounts of both, phenylpropanoids and flavonols were higher in Eastern type, on average, by 27 and 20.8 %, respectively. These two carrot types did not differ with respect to the anthocyanin content, which was very low. As RSA was highly correlated with the content of phenolics, and particularly with phenylpropanoids, antiradical activity measured for Eastern carrots was also higher than for Western type. However, Eastern carrots showed 1.6 times higher antiradical capacity than Western carrots did that is much more than expected from the higher amounts of phenolic compounds. This may suggests that different qualitative composition of phenolic compounds in carrot belong to these two types. Among Eastern carrots, the most valuable seem accessions developing red roots, in particular Asian landraces ‘Gajar’, ‘Pusa Kesar’ and ‘Shahpur Special’, but not a modern, red cultivar ‘Nutrired’. These accessions possessed much higher (1.5 times) content of all phenolics and antiradical activity in the first year of the study. In 2009, the total rainfall was two-times less than in 2010; thus, it may indicate that the red carrots of Asian origin respond to water deficiency stress by increased biosynthesis of phenolic compounds. Two breeding populations (BL-JKI-4 and BL-JKI-5) of Asian origin and developing orange roots were also distinguished by 2–3 times higher content of phenolics and RSA than most other orange accessions. In a group of white and yellow accessions, only Asian ‘Shima Ninjin’, ‘Mestnaya’ landrace and its selections (Mestnaya-S1-p and Mestnaya-S1-y) exceeded other accessions, including modern cultivars classified to Eastern gene pool. Finally, the most distinctive were two cultivars, ‘Deep Purple’ not assessed in SSR analysis and ‘Anthonina’ classified initially to Western carrot type. Both cultivars had roots with intense purple color suggesting that parental materials from Asia were used for their creation. Therefore, both purple carrots have Asian genetic background.

**Table 5:** The content of phenolic compounds [mg 100 g^−1^ FW] and RSA [%] in Eastern and Western carrot types (purple carrots are excluded from comparison)

Carrot type	Total phenols (Folin–Ciocalteu)	Total phenols (UV/Vis)	Phenylpropanoids	Flavonols	Anthocyanins	RSA
Eastern	30.8 a^a^	40.2 a	9.4 a	6.4 a	0.5 a	8.5 a
Western	20.1 b	32.4 b	7.4 b	5.3 b	0.4 a	5.3 b

^a^Means followed by the same letter in each column do not differ significantly according to *t*-test at *p* = 0.05

The fact that Eastern carrots differ in compound composition from Western type has been also recently shown for other constituents. The comparison of 118 carrot accessions showed that European and American accessions (presumably mostly belonging to Western gene pool) had about 18 % more sugars than Asian accessions (presumably mostly belonging to Eastern gene pool) and the difference was observed for advanced cultivars as well as for landraces, and also when only orange rooted accessions were compared. Also, European orange accessions were richer in carotenoids than those originating from Asia by 25 %. Moreover, advanced cultivars had more carotenoids than landraces that demonstrates quantitative changes in chemical composition as a result of breeding progress [2].

## Conclusions

Carrot is one of the most important vegetables in the world, its bioactive constituents may be beneficial to a vast number of consumers. Western carrot type, mainly with orange roots, dominates in production and only recently new cultivars characterized by yellow, red or purple roots have been introduced in America and Europe while in Asia such landraces and cultivars are common. The results presented here demonstrate that within carrot genetic resources, a wide diversity of antioxidant phenolics exists. Carrots developing purple roots that are rich in anthocyanins seem to be exceptionally valuable, but red carrots, although almost free of anthocyanins, possess higher antioxidant capacity than orange carrots. The screening of the carrot collection comprising accessions of different origin indicates that Eastern carrot gene pool may be a source of genes important for breeding new modern cultivars of high quality. Unfortunately, carrots of Eastern type are not suitable for direct implementation of production in temperate climate and have many undesired characters like the tendency for early bolting, not cylindrical root shape and usually rough skin. They are also more susceptible to several pathogens and pests occurring on the Western hemisphere. Therefore, the development of high quality cultivars with the use of Eastern type and suitable for commercial production can be achieved only after intensive breeding efforts. However, usually higher levels of phenolics found in landraces than in modern cultivars may suggest that the content of phenolics, thus antioxidant capacity, may be negatively correlated with agronomical traits making breeding progress difficult.

## Electronic supplementary material

Below is the link to the electronic supplementary material.

Online Resource 1 Plant material (PDF 36 kb)

## Abbreviations

ABTS: 2,2′-azino-bis (3-ethylbenzothiazoline-6-sulphonic acid)
DPPH: 1,1-diphenyl-2-picrylhydrazyl radical
RSA: Radical scavenging activity
SSR: Simple sequence repeat (microsatellite) DNA marker
