# Supplementary material for: The Content of Phenolic Compounds and Radical Scavenging Activity Varies with Carrot Origin and Root Color
Source: Plant Foods Hum Nutr. 2013 Apr 24;68(2):163–70. doi: 10.1007/s11130-013-0351-3 (PMC3659275; doi:10.1007/s11130-013-0351-3)
Supplement: Supplementary file 1 — Plant material (PDF 36 kb) [file 11130_2013_351_MOESM1_ESM.pdf]

Online Resource 1. Plant material

No.	Root color	FS No.	Accession Name	Source[a]/No.	Country	Origin	Type[b]	Status[c]
1.	orange	FS_43	Amsterdam 3	Commercial	POL	Europe	W	OP
2.	orange	FS_50	BL-CR2-6032	Satimex / 6032	DEU	Europe	W	BP
3.	orange	FS_70	BL-JKI-4	JKI	IND	Asia	E	BP
4.	orange	FS_71	BL-JKI-5	JKI	IND	Asia	E	BP
5.	orange	FS_49	Finezja	Spojnia	POL	Europe	W	OP
6.	orange	FS_72	Garga Serk	WGRU / 10264	PAK	Asia	E	LR
7.	orange	FS_42	Hekinan Senko 5sun	MKS	JPN	JPN	W	OP
8.	orange	FS_39	Kokubu Senko Oonaga	MKS	JPN	JPN	E	OP
9.	orange	FS_74	Long Red	WGRU / 10246	ETH	Africa	E	LR
10.	orange	FS_36	Nantes Apollo	NGB	DNK	Europe	W	OP
11.	orange	FS_06	Nerac	Bejo	NLD	Europe	W	F1
12.	orange	FS_75	Red Carrot	WGRU / 10355	CHN	Asia	E	LR
13.	orange	FS_05	Santa Cruz	Seminis	USA	USA	W	OP
14.	orange	FS_40	Sapporo Futo	MKS	JPN	JPN	W	OP
15.	orange	FS_69	Stratova	WGRU / 10626	CSK	Europe	W	OP
16.	purple	FS_09	Anthonina	Seminis	USA	USA	W	OP
17.	purple	FS_07	Deep Purple	Bejo	NLD	Europe	E	F1
18.	red	FS_47	Gajar	WGRU / 10146	PAK	Asia	E	LR
19.	red	FS_73	Kintoki	WGRU / 7174	JPN	JPN	E	LR
20.	red	FS_04	Nutrired	Seminis	USA	USA	E	OP
21.	red	FS_22	Pusa Kesar	WGRU / 6755	IND	Asia	E	LR
22.	red	FS_38	Shahpur Special	WGRU / 6752	IND	Asia	E	LR
23.	white	FS_11	Blanche 1/2 longue des vosges	INH	FRA	Europe	W	OP
24.	white	FS_10	Kuettiger	JKI	CHE	Europe	W	OP
25.	white	FS_64	Mestnaya	WGRU / 13405	RUS	Asia	E	LR
26.	white	FS_45	White Belgian	WGRU / 8720	GBR	Europe	W	OP
27.	white	FS_01	White Satin	Bejo	NLD	Europe	W	F1
28.	yellow	FS_03	BL-710015	Seminis	USA	USA	W	BP
29.	yellow	FS_37	China Yellow	MKS	CHN	Asia	E	OP
30.	yellow	FS_46	Lobbericher	Commercial	DEU	Europe	W	OP
31.	yellow	FS_68	Mestnaya-S2-p	WGRU / 13405	RUS	Asia	E	LR
32.	yellow	FS_67	Mestnaya-S1-y	WGRU / 13405	RUS	Asia	E	LR
33.	yellow	FS_41	Shima Ninjin	MKS	JPN	JPN	E	OP
34.	yellow	FS_63	Yellow Belgian	DAU	NLD	Europe	W	OP
35.	yellow	FS_02	Yellowstone	Bejo	NLD	Europe	E	OP

[a] Source: Bejo – Bejo Zaden B.V., Warmenhuizen, Netherlands; DAU – Leibniz Institute of Plant Genetics and Crop Plant Research, Gatersleben, Germany; INH – Institut National d'Horticulture et de Paysage, Angers, France; JKI – Julius Kühn Institute, Quedlinburg, Germany; MKS – Mikado Kyowa Seed Co. Ltd., Chosei, Japan; NGB – Nordic Genetic Center, Alnarp, Sweden; Satimex – Satimex Quedlinburg Handelsgesellschaft GmbH, Quedlinburg, Germany; Spojnia – Spójnia Hodowla i Nasiennictwo Ogrodnicze Ltd. Nochowo, Poland; WGRU – Warwick Genetic Resources Unit, Warwick University, Wellesbourne, England

[b] Type: Accessions were classified to either the Eastern (E) or Western (W) type according to DNA analysis of simple sequence repeats (Baranski, Maksylewicz-Kaul, Nothnagel, Cavagnaro, Simon & Grzebelus, 2012) or the accession origin

[c] Status: BP – breeding population; OP – open pollinated cultivar; F1 – hybrid; LR - landrace
